# Correspondence

**Published:** 1888-09

**Authors:** W. Mitchell


### CORRESPONDENCE.

To the EDITOR OF THE DENTAL REGISTER,

*Dear Sir:*—Perhaps a few items as to the condition of our profession on this side of the Atlantic would not prove uninteresting to your readers; so I will try and present a few points for their consideration.

When there is a demand for any commodity, that commodity is usually forthcoming, even in this conservative country, and as the necessity for experience in advanced methods of practice by the rank and file of the profession here was manifest, the two Dental Hospitals of London instituted post-graduate courses. I could but note the difference between the methods here and those in vogue in the United States in those institutions where post-graduate courses are a feature, here, the instruction is almost exclusively of a mechanical or manipulative nature, while there, it is in the nature of advanced scientific work, in both cases undoubtedly aiming in the right direction, for in the United States the tendency is toward operative perfection, while here, a paucity of technical lore is supposed to meet nearly all requirements.

In looking over the subjects considered, we find some of the instructors persons of ripe experience, and whose lectures were a treat to listen to, but in looking through some of the journals a smile is provoked when we learn at this late day there are those of years of experience who require instruction in the application and use of the rubber dam, and mention is also made of the fact that one of the lecturers explained the Electric Mallet, "and actually filled a crown cavity in the presence of the class," thus demonstrating its utility. Such a state of affairs as this may seem amusing to those in the habit of witnessing clinics on the other side, but clinics here, as an adjunct to dental meetings is something almost unheard of, and where attempted, the most scant arrangements are made to promote the success of the work arranged for by the demonstrator. This can only be accounted for in one of two ways, either a general lack of capacity, or the supposed possession of an idea that must be made to conduce entirely to personal ends, in the latter case I can readily understand why it is essential to closely guard that idea, it may be the only one its possessor ever had.

The profession here are greatly indebted to Dr. W. St. George Elliott, and Mr. Gaddes, the Dean of the National Dental Hospital, who by their careful and exacting methods with their students have created quite a new order of things, having, as instructors, demonstrated the possibility of getting out of a rut of indifference into which dental education had fallen, by demanding a more intelligent study of individual cases, and the application of recent methods and appliances instead of fostering obsolete and antiquated ideas.

I understand the National Dental Hospital will soon enlarge its premises and at the same time its capacity for carrying on the work for which it now bears such a good reputation.

For some time past there has been much correspondence and editorial space devoted to a discussion and criticism of the qualifications and degrees necessary to enable the dentist to best conserve the interests of his patients, and fully establish himself as a member of a learned and respected profession. Of course, in the comparisons that are drawn the American degrees come in for some very unfavorable comment and criticism; the arguments are just about as logical as those used some time ago by the medical journals here when commenting upon the American medical degree, one argument used was, "The D.D.S. was a peculiar designation" possibly so, almost any thing that is original, and vested with an air of independence, is "peculiar" to the average Englishman, and while here they may claim priority in medical matters, common courtesy would suggest that in dental matters this country has still a few things to learn before they should pose as dictators as to the course American dental colleges should pursue. If "brevity is the soul of wit," in its dental and medical degrees the United States maintains an enviable position, with its D.D.S. and M.D. as compared with the L.D.S. and R.D.S.—which by the way are no degrees at all—simply licences—the L.R.C.P., M.R.C.S., etc., etc,, of course there is nothing fulsome, or suggestive of flatus about the degrees attainable here when you look at it through the large end of the telescope, but modesty is a virtue that editors should possess when drawing comparisons, but possibly it never occurred to them that nearly every subject possesses two sides.

There is no doubt but a great many evils have existed, and in some cases still exist in American colleges, but the example already set by some will produce a salutary effect upon the profession at large, the disreputable methods resorted to by some institutions in their scramble for patronage have in many instances trailed is the dust of execration institutions whose names should have been a power, instead of a curse among American Dental Colleges. Europe to quite an extent has been a dumping ground of the products of bogus or ill-conditioned institutions, the effect of which is keenly felt by those who have tried to practice as they preached, and to the pioneers of the American contingent of the profession here all credit is due for upholding the dignity of their profession when it has been assailed through the obliquity and disreputable methods of a class of adventurers whose professional standing at home and abroad has ever been at zero.

A state of affairs has long existed here that has brought reproach upon our profession, and done much to modify the appreciation which American operators have hitherto enjoyed; many young men just leaving college, and with undefined plans for the future have been lured by the temptations incidental to "practicing in Europe," and have come over, only to be woefully deceived as to the position they would occupy, and to find the frigid scalpula of their professional brethren here presented at every turn, a condition of things that does not conduce to the harmony that should exist between those whose aims and endeavors should be a unit.

If the American Dental Association of Europe, or the Odontological Society of Great Britain could see their way clear when called upon to give the professional standing of persons here desiring assistance, a mutual advantage would ensue that would be productive of much good. Of course I fully understand where an American assistant is engaged, an English one must necessarily seek an engagement elsewhere, in this it is a case of the "survival of the fittest," and whichever proves himself most worthy and competent to him should preference be shown. Our profession is not alone in possessing a certain class who for a little immediate preferment will sacrifice ulterior possibilities, and it is to this class instructors could with great benefit incorporate in their practical lectures the ethical phase of Dentistry.

In the post-graduate course of the National Dental Hospital spoken of in the opening passages of this letter, Dr. W. St. George Elliott, lectured upon and demonstrated "Gold Crown" work; Walter Coffin lectured upon and explained the system of regulating that has made the name of father and son noted the world over.

Dr. Cunningham, of Cambridge, discoursed upon the "Immediate Method of Filling Roots," a line of practice that is destined to modify to quite an extent future practice. Messrs. Rose and Humby demonstrated the use of a steam swager for producing metal models and counters between which to produce contour vulcanite work. This was an exceedingly practical and useful affair, and it is a source of surprise to me why the profession at large is not more conversant with a method conducive to such happy results.

Implantation has also received attention; this at the hands of Dr. Cunningham. His cases show a favorable per cent. of success: time though will demonstrate whether this sanguinary and painful operation is a justifiable one or not. Dr. Cunningham takes issue with Dr. Younger against the revitilization of the pericementum, in the case of implantation of dry teeth, and favors the use of teeth in a fresh state, with pericementum as intact as possible. This seems to me to be tenable ground to occupy, but for those enthusiasts who claim that pulp circulation has been established after implantation, I think this exists only in their imagination. Yours Truly,

W. Mitchell.

---

It is reported that the dentists are taking the stump for revenue only all over the country. Politicians will have no patients with this sort of thing.—*Phila. Ledger.*

